# Biofilm Induced Tolerance towards Antimicrobial Peptides

**DOI:** 10.1371/journal.pone.0001891

**Published:** 2008-04-02

**Authors:** Anders Folkesson, Janus A. J. Haagensen, Claudia Zampaloni, Claus Sternberg, Søren Molin

**Affiliations:** 1 Infection Microbiology Group, BioCentrum-DTU, Technical University of Denmark, Lyngby, Denmark; 2 Department of Molecular Cellular Animal Biology, University of Camerino, Camerino, Italy; Massachusetts General Hospital, United States of America

## Abstract

Increased tolerance to antimicrobial agents is thought to be an important feature of microbes growing in biofilms. We address the question of how biofilm organization affects antibiotic susceptibility. We established *Escherichia coli* biofilms with differential structural organization due to the presence of IncF plasmids expressing altered forms of the transfer pili in two different biofilm model systems. The mature biofilms were subsequently treated with two antibiotics with different molecular targets, the peptide antibiotic colistin and the fluoroquinolone ciprofloxacin. The dynamics of microbial killing were monitored by viable count determination, and confocal laser microscopy. Strains forming structurally organized biofilms show an increased bacterial survival when challenged with colistin, compared to strains forming unstructured biofilms. The increased survival is due to genetically regulated tolerant subpopulation formation and not caused by a general biofilm property. No significant difference in survival was detected when the strains were challenged with ciprofloxacin. Our data show that biofilm formation confers increased colistin tolerance to cells within the biofilm structure, but the protection is conditional being dependent on the structural organization of the biofilm, and the induction of specific tolerance mechanisms.

## Introduction

Natural conjugative plasmids express factors that can stimulate planktonic bacteria to form biofilm communities [1]. Biofilm formation also favors the infectious transfer of plasmids [2], [3], and many medically relevant microbial pathogens are often associated with a variety of conjugative plasmids indicating an increased tendency for biofilm formation [1], [4]–[6]. Microbial biofilms are notoriously hard to treat with antimicrobial compounds. The molecular nature of this apparent resistance is not well elucidated and a number of mechanisms have been proposed to explain the reduced susceptibility [7]. The architecture of mature biofilms varies considerably from flat homogenous layers of cells to highly organized cell clusters characterized by mushroom shaped structures interspersed by water-filled channels [8]. The organization of the biofilm affects the distribution of chemical and physiological gradients within the microbial assembly, which in turn affects the metabolic state of the bacteria, resulting in a heterogeneous congregation of cells in different physiological states [8]–[10]. The spatial and physiological heterogeneity of the biofilm architecture has the potential to affect the antimicrobial tolerance profiles of the organisms in the biofilm by forming microhabitats with diverse properties [11].

*Escherichia coli* (*E.coli*) K12 strains usually form unstructured flat biofilms in comparison with the highly organized and substantial biofilms formed by bacteria such as *Pseudomonas aeruginosa* (*P.aeruginosa*) [6], [12]. Introduction of transfer constitutive IncF plasmids dramatically changes the organization of *E.coli* biofilms to a structure resembling those reported for *P.aeruginosa* [1], [6]. Biofilm organization is determined by the configuration of the transfer pili since various mutants affected in the processing and activity of the pili exhibit differentially structured biofilms [6].

The ability to survive exposure to cationic antimicrobial peptides is an essential virulence property for many microbial pathogens [13]. Antimicrobial peptides are an integral part of the host defense system and believed to kill microorganisms by affecting their membrane integrity. Colistin is an amphipathic polypetide antibiotic utilized in the treatment of multi-resistant *P.aeruginosa* causing chronic pulmonary infections among cystic fibrosis patients. The peptide antibiotic is also extensively used both as feed additive and in the treatment of severe colibacillosis caused by pathogenic *E.coli* in livestock [14], [15]. Resistance towards antimicrobial peptides such as colistin is seldom identified in the clinical setting [16]. Gram-negative bacteria have evolved mechanisms that remodel the composition of the outer membrane through modification of the lipopolysaccharide (LPS) molecules [13]. The modifications lead to an increased tolerance to antimicrobial peptide exposure, and is controlled by a two-component regulatory system encoded by homologs to the *Salmonella typhimurium pmrA/B* genes in many bacterial species [17], [18]. LPS alterations are thought to play a significant role during infection by conferring increased bacterial tolerance to host antimicrobial factors and avoidance of immune system recognition [13].

In contrast to colistin, the primary *E.coli* target for fluoroquinolone antibiotics such as ciprofloxacin is the topoisomerase II enzyme, DNA gyrase, which is essential for DNA synthesis [19]. Resistance to fluoroquinolones in *E. coli* occurs by chromosomal mutation and requires alterations in either of the genes that encode the topoisomerases (*gyrA* and *gyrB*, encoding gyrase, and *parC* and *parE,* encoding topoisomerase IV) or in genes that influence cell permeability or drug export [20], [21].

In this report, we challenge *E.coli* biofilms with differential structural organization with colistin or ciprofloxacin, two clinically relevant antibiotics with different molecular targets and mechanisms of resistance. We show that biofilm formation increases the survival of the microbes after challenge by the peptide antibiotic but not by the fluoroquinolone. The increased survival is dependent on the *basR/basS* (*pmrA*/B) two-component system, since disruption of their encoding genes abolishes the observed protective effect while not affecting the organization of the biofilm. We demonstrate that the increased survival is due to development of a tolerant subpopulation within the biofilm and that the formation of the subpopulation is influenced by the spatial organization of the biofilm indicating that intra-biofilm physiochemical gradients promote antibiotic tolerance within spatially structured environments.

## Results

### *E.coli* cells in biofilms differing in organization display differential survival after colistin treatment

**Figure 1:**
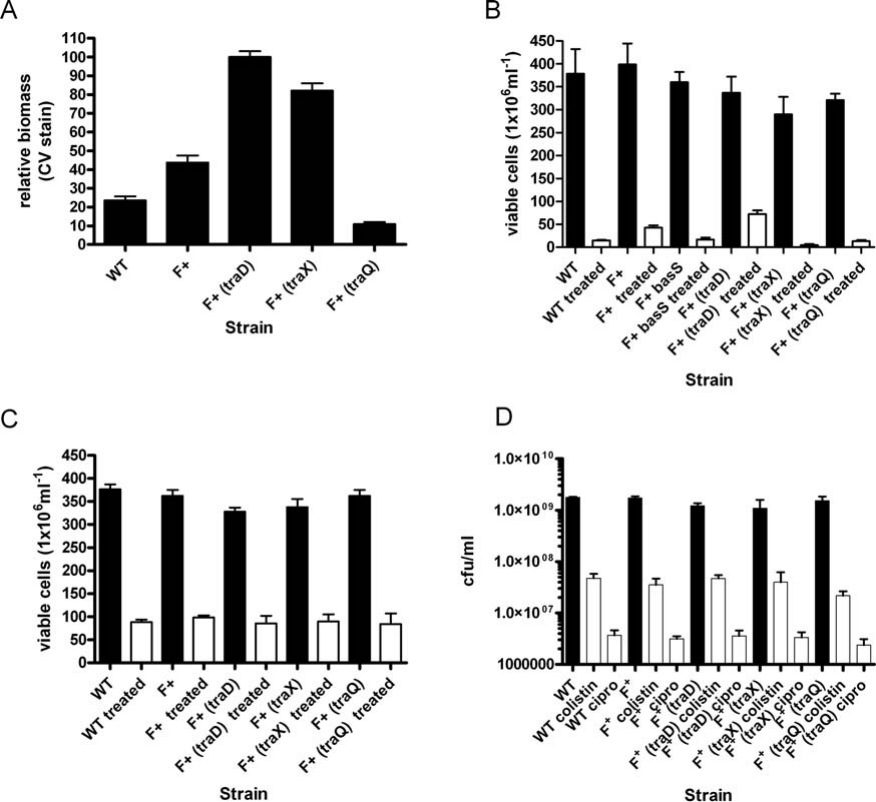
* E.coli* cells in biofilms differing in organization display differential survival after colistin treatment. A). Microtitre plate biofilm assay with the parental *E. coli* SAR18 in the presence and absence of the de-repressed IncF plasmid and mutants in the IncF plasmid (*traD, traX,* and *traQ*,) stained with crystal violet. Values are normalized with respect to the mean of SAR18 F^+^ (*traD*) set as 100% B). A microtitre plate biofilm antibiotic tolerance assay, in which the indicated strains were challenged with 10 µg/ml colistin for 24h and bacterial survival determined by viable count as indicated by white bars. Untreated control is indicted by black bars. C). Microtitre plate biofilm antibiotic tolerance assay, in which the indicated strains were challenged with 0.3 µg/ml ciprofloxacin for 24h and bacterial survival determined by viable count as indicted by white bars. Untreated control is indicated by black bars. D). The survival of planktonically cultured stationary phase *E. coli* SAR18 in the presence and absence of the de-repressed IncF plasmid and mutants in the IncF plasmid (*traD, traX,* and *traQ*,) after a 24 h 10 µg/ml colistin or 0.3 µg/ml ciprofloxacin challenge. Black bars indicate CFU in untreated culture while white bars indicate treated cells as specified in the figure.

The presence of transfer constitutive IncF plasmids fundamentally influences the architecture and organization of *E.coli* biofilms [6]. The final spatial arrangement of the biofilm is determined by the configuration of the transfer pili, as various mutants in the processing and activity of the organelle display differentially organized biofilms [6]. F pilus synthesis requires 13 essential genes and 4 auxiliary genes of plasmid origin. TraQ is required for insertion of propilin (TraA) in the inner membrane. Absence of the TraQ chaperone results in rapid degradation of TraA in the cytoplasm [22]. TraX is responsible for acetylation of the N-terminus of mature pilin, but the modification is non-essential for pilus elaboration and transfer proficiency [23], [24]. TraD is not involved in pilus assembly but instead has an essential role in DNA transfer at a stage following cell/cell contact [25]. To study the effects of differential biofilm organization in relation to antibiotic tolerance, we established a static biofilm and survival assay of wildtype, *traQ*, *traX*, and *traD* mutant derivatives of pOX38 [26] in *E.coli* K12 CSH26 derivative SAR18, which were compared with biofilms formed by plasmid-free SAR18 in standard flat-bottomed 96 well microtiter dishes. The strains were inoculated in Luria broth and incubated statically for 24h. Differential biomass accumulation for SAR18 F^+^ and the various mutants compared to SAR18 was confirmed by crystal violet staining of the wells (Fig. 1A). The results were consistent with the previously observed biofilm phenotype in the flow-chamber biofilm system. SAR18F^+^, F^+^(*traD*) and F^+^(*traX*) accumulate increased amounts of biomass in microtiter wells compared to SAR18F^+^(*traQ*) and SAR18 lacking F^+^ (Fig. 1A) [6]. For the colistin survival assay, the established biofilms were challenged with 10 µg/ml of the cationic antimicrobial peptide colistin instead of washing and crystal violet staining [27]. The killing activity and mode of action of antimicrobial peptides is highly influenced by experimental conditions and bacterial cell number [28].To keep the experimental conditions as comparable as possible the antibiotic was added directly to the wells without prior washing and removal of putative planktonic cells. After an additional 24h incubation with antibiotic, the total surviving CFU were determined by serial dilution and plating on culture media by dislodging the biofilms from the microtiter wells by vigorous aspiration. We observed a three fold difference in survival between SAR18 and SAR18 F^+^, where the surviving CFU were significantly elevated in SAR18 F^+^ biofilms (Fig. 1B) (P<0.0044). Survival was even more enhanced in the biofilm produced by the *traD* mutant where the difference was five fold compared to SAR18 (Fig. 1B). Survival did not correlate with the amount of biofilm biomass formed because the *traX* mutant, that forms biofilm in a quantity approximately equal to the *traD* mutant, did not show any enhanced survival (Fig. 1B). The presence of the F derivatives did not affect colony forming unit (CFU) formation in the wells since no difference in CFU counts could be detected in untreated control wells (Fig. 1B and C) (P>0.05). Addition of the fluoroquinolone ciprofloxacin (0.3 µg/ml), an antibiotic with an intracellular target, showed no significant difference in killing of the different strains (Fig. 1C) (P>0.05). The MIC for colistin and ciprofloxacin were 1 µg/ml and 0.03 µg/ml, respectively, for all of the strains in the study.

### Mutations in the transfer operons do not confer protection in plantonic cultures

Mutations in the transfer operon may have important pleiotropic consequences independent of the biofilm context, we therefore investigated the survival after colistin and ciprofloxacin treatment of planktonic cultures to the of wildtype, *traQ*, *traX*, and *traD* mutant derivatives of pOX38 and plasmid-free SAR18 biofilm cultures. The strains were inoculated in Luria broth using standard polystyrene culture tubes and incubated under shaking (rpm 220) conditions for 24 h. The cultures were then challenged with 10 µg/ml colistin for an additional 24h and surviving colony-forming units (CFU) were determined by serial dilution and plating on culture media. For all strains approximately 3×10^7^ CFU had survived the colistin challenged, while the untreated control cultures had reached a CFU of 1×10^9^. There was no significant (P>0.05) difference in survival between wildtype, *traQ*, *traX*, and *traD* mutant derivatives of pOX38 and plasmid-free SAR18 biofilm cultures (Fig. 1D). If the shaken over night cultures instead were challenge with 0.3 µg/ml ciprofloxacin for 24 h, 2×10^6^ CFU were detected corresponding to a 3 log difference of survival between the different growth model systems (Fig. 1D). These data indicate that the presence of the F plasmid and its mutant derivatives does not affect antibiotic tolerance during planktonic growth.

### The increased survival of cells in colistin treated biofilms is dependent on a functional *basS* gene

**Figure 2:**
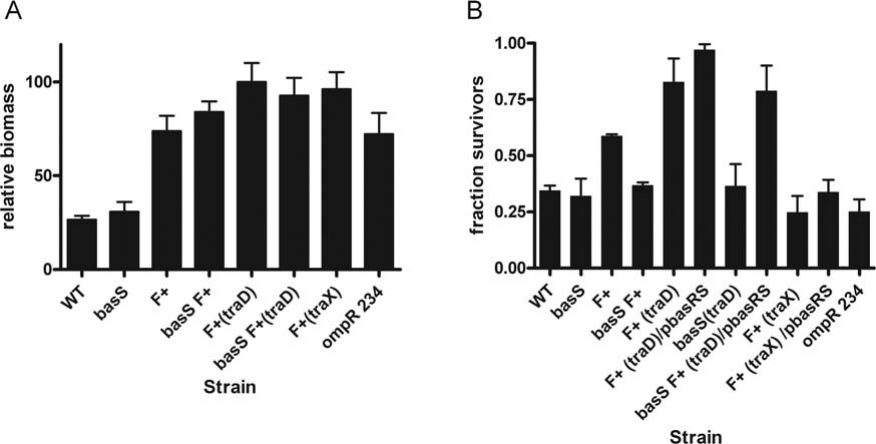
The increased survival of cells in biofilms is dependent on a functional *basS* gene. A). Microtitre plate biofilm assay with the parental *E. coli* AF504 in the presence and absence of the de-repressed IncF plasmid and mutants in the IncF plasmid (*traD,*), insertion deletions of the *basS* and *basR* genes, and the curli overproducing *opmR* strain stained with crystal violet. Values are normalized with respect to the mean of AF504 F^+^ (*traD*) set as 100%. B). A microtitre plate biofilm antibiotic tolerance assay in which the indicated strains were challenged with 10 µg/ml colistin for 24 h and bacterial survival determined. The fraction untreated/treated is indicated for each strain.

The *E.coli* K12 strain MG1655 is better characterized genetically than the SAR18 strain. Moreover, SAR18 harbors a number of antibiotic resistance markers that make genetic manipulation difficult (Table 1). We included the strain AF504, a nalidixic acid resistant derivative of MG1655, and a curli over-producing *ompR234* mutant of MG1655 in the investigation. Curli overproduction results in increased biofilm formation in the biofilm flow chamber system in an F-plasmid independent manner [6]. To investigate if the observed increase in colistin tolerance of biofilm cells is genetically determined or due to a generic difference in biofilm permeability, we introduced insertion deletions into the *basS* gene of AF504, using one-step allelic replacement [29]. We established biofilms of these mutant strains and compared the survival of the bacteria with or without the F-plasmid and its mutant *traD* variant after colistin treatment. As expected, introduction of the F-plasmid or its *traD* mutant derivative into AF504 drastically increased biofilm formation as well as colistin tolerance of the resulting strains (Fig. 2A and B). The protective effect of biofilm formation with F^+^ and F^+^(*traD*) was completely abolished in the *basS* mutants, and the survival reached a level comparable to the survival of plasmid free AF504 (Fig. 2B). If the *basR* and *basS* genes were reintroduced on a multicopy plasmid into the *basS traD* mutant biofilm protection was restored (Fig. 2B), while if the same plasmid was introduced into AF504 or AF504 F^+^(*traX*) no effect on survival could be detected (Fig. 2B). If the vector alone was introduced to the strains, no effect on biofilm formation or survival could be detected (data not shown). The nalidixic acid resistance of AF504 does not affect the outcome of the biofilm survival assay since there is no detectable difference in survival between MG1655 and AF504 after colistin challenge (data not shown). These results indicate that a specific genetic mechanism is underlying the observed increased tolerance in biofilm forming *E.coli*. Over-expression of the curli fimbriae drastically influences the biofilm development in *E.coli* and such strains form biofilms comparable to the ones formed by F^+^(*traD*) and F^+^(*traX*) mutants (Fig. 2A [6], [30]. No increase in survival after colistin treatment compared to the plasmid free AF504 was detected, demonstrating that biofilm formation and survival after antibiotic treatment are not directly correlated (Fig. 2B). If the same *basS* mutation was introduced into Sar18 F^+^, pili mediated protection was abolished, showing that *basS* dependence is not exclusive for AF504 (Fig. 1B). Increased concentrations of colistin decreased the number of viable cells within the microtiter wells in a concentration dependent manner. Colistin concentrations above 60 µg/ml reduced the number of viable cells to below the detection level of 100 CFU/ml (Data not shown). The MIC for colistin for all of the AF504 derived strains were 1 µg/ml in microtiter assay.

**Table 1:** Bacterial strains used in this study.

Strains	Description	Reference
SAR18	CSH26 *attB*::bla-PA1/04/03gfpmut3*Cm^r^	[6]
AF504	MG1655 Nal^r^ *ilvG, rfb-50, rph-1*	This work.
AF504 *gfp*	AF504 *attB*::*bla-rrnB*P1::*gfp*mut3b*	This work
AF574	AF504 *basS::cm*	This work
AF518	AF504 *basS::kan*	This work
AF574 *gfp*	AF504 *basS::cm attB*::*bla-rrnB*P1::*gfp*mut3b*	This work
PHL 628	MG1655 *ompR234*	[63]
PHL 628 *gfp*	PHL628 *ompR234 attB*::*bla-rrnB*P1::*gfp*mut3b*	This work
XL10-Gold	*TetrΔ (mcrA)183 Δ(mcrCB-hsdSMR-mrr)173 endA1 supE44 thi-1 recA1gyrA96 relA1 lac Hte [F′ proAB lacIqZΔM15 Tn10 (Tetr) Amy Camr*]	Stratagene

### *E.coli* flow-chamber biofilms are sensitive to the cationic antimicrobial peptide colistin

**Figure 3:**
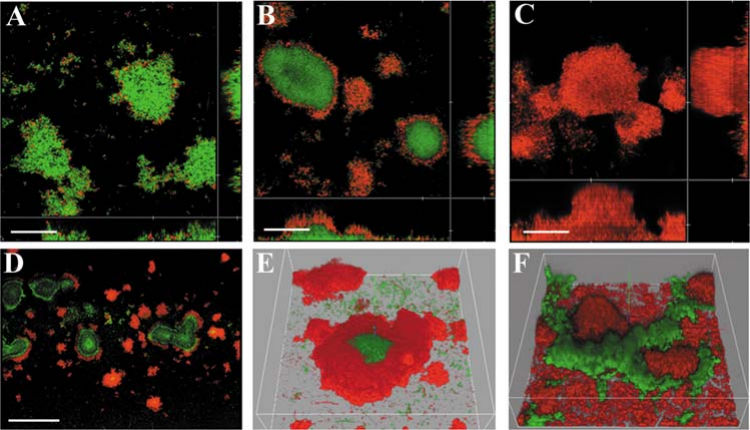
A colistin tolerant subpopulation is formed in mature *E .coli* flow-chamber biofilms. CLSM micrographs of a mature AF504 *gfp*/F^+^ biofilm after A) control, B) 6.5h; and C) 24h 25 µg/ml of colistin challenge. The biofilm was stained with PI at the specified time points before image acquisition. Scale bars represent 50 µm D) CLSM micrograph showing the location of live (green) and dead (red) cells in *E. coli* AF504 *gfp/*F^+^ biofilm grown for 48 h and then exposed to 10 µg/ml of colistin for 24 h, an overview image taken with a 10× objective. Scale bar represent 300 µm. E) CLSM 3D micrograph image taken with a 40× objective of a single microcolony in the AF504 *gfp/*F^+^ biofilm. F) CLSM micrograph 3D structure showing the spatial distribution of living (green) and dead (red) cells of *E. coli* AF504 *gfp/*F^+^ in a 6 days old biofilm, that has been grown in antibiotic free media for 2 days and then exposed to 25 µg/ml colistin for 24 h and then switched back to antibiotic free medium.

To be able to study biofilm formation *in vitro* in the hydrodynamic continuous-flow biofilm model system [31] we tagged AF504 F^+^ with the green fluorescent protein (GFP) under the control of a ribosomal promoter forming the strain AF504 *gfp* F^+^. The flow chambers were seeded with the strain and incubated for 48h at 30°C in glucose minimal medium. The mature biofilms were then exposed to 25 µg/ml colistin for 0h (untreated control), 6.5h and 24h and stained with propidium iodide (PI) to distinguish living GFP expressing cells and dead PI stained cells (Fig. 3 A, B and C). The untreated control biofilm exhibited a few dead (red) cells spread out through the biofilm (Fig. 3A) while the majority of the cells in the treated biofilm were clearly dead after 24 h (Fig. 3 C). The killing activity of the colistin moved from the edge of the microcolonies to the inner part as shown in time series image capturing (Movie S1). The time series also confirmed that detachment was absent or low under these conditions. To determine the proportion of viable cells in the biofilm channels, the biofilm was dislodged from the flow-channel and the CFU/ml were determined and normalized by the OD450 value obtained for each channel. The number of viable cells recovered from the channel decreased from 10^9^ to 10^3^ CFU/OD_450_ in 24 hours of treatment and reached 10^2^ CFU/OD_450_ after 48 hours of drug-exposure. The number of viable cells in the control channel remained constant during the time of the experiment (10^9^ CFU/OD_450_). If 50 µg/ml colistin was used, an exposure for 24 h was enough for complete eradication of the biofilm and indeed no growth was observable after plating. If plasmid free AF504 *gfp* cells were inoculated in the flow channel system a few scattered loosely attached cells could be detected through the channel after 24 h and after a 24 h, 25 µg/ml colistin challenge very few bacteria could be detected (data not shown).

### A colistin tolerant subpopulation is formed in mature *E. coli* flow-chamber biofilms

*E. coli* mature biofilm cells are killed efficiently by colistin through gradual penetration into the centre of the microcolonies (Fig 3 A, B and C). However, in large microcolonies we observed a surviving population gradually increasing in biomass during the first 48 h after addition of colistin. This phenomenon was observed using 10 µg/ml and 25 µg/ml colistin (Fig. 3D and E). The tolerant subpopulation only formed in fully mature biofilms of bacteria expressing the transfer pili and was able to re-develop into a new biofilm if colistin free media was supplied to the flow chamber system (Fig. 3F). One day after a shift from 25 µg/ml colistin to antibiotic-free medium the tolerant population was growing and after additional two days, microcolony formation and biofilm re-development was observed (Fig. 3F).

### Colistin tolerance is not due to resistance mutations

Based on these findings we wanted to investigate if the developing colistin tolerant subpopulations were the results of genetically mutated resistant cells, or if they represented phenotypically tolerant variants. We isolated the biofilm material from 2-day-old flow channel biofilms, grown in glucose minimal media, which had subsequently been exposed to 10 µg/ml or 25 µg/ml colistin, for 24–48 h. The isolated biofilm samples were homogenized and stained with PI after which individual cells were sorted with a FACS cell sorter in red and green subpopulations. The red population was not viable as shown by plating on LB plates whereas the green population was growing on LB plates, but not on plates containing colistin (10 µg/ml or 25 µg/ml). Development of a tolerant subpopulation in the biofilm after treatment with colistin thus seems to be linked to adaptation or activation of a surviving phenotype suggesting that a transiently colistin tolerant subpopulation is formed in the biofilm. When the cells were isolated from the flow chamber and re-grown without colistin they lost the tolerance to the peptide and became sensitive to the antibiotic, showing that the surviving tolerant cells were not genetically resistant mutants selected in the flow chamber.

### Colistin tolerance is correlated to biofilm structure and architecture

**Figure 4:**
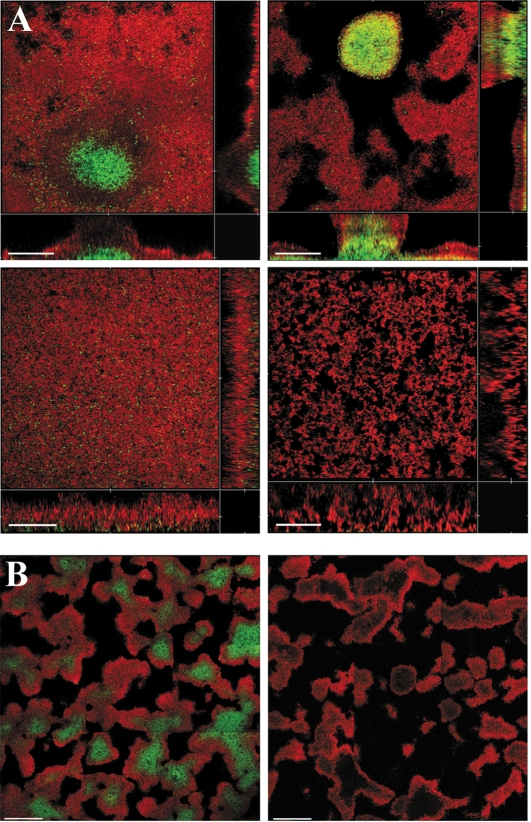
The colistin tolerance is influenced by biofilm structural organization and tolerance induction is *basS* dependent. A). CLSM micrographs showing the distribution of live and dead cells in biofilms of the SAR18/F^+^ (top left panel), SAR18/F^+^(traD) (top right panel), SAR18/F^+^(traX) (bottom left panel), and PHL628 *gfp ompR234* (bottom right panel) after 24h challenge by 10 µg/ml colistin. Scale bars represent 50 µm. B). CLSM micrographs showing the distribution of live and dead cells in biofilms of AF504 *gfp*/F^+^ (left panel) and AF574 *gfp basS*::Kan/F^+^ (right panel) mutant showing the *basS* dependence of subpopulation formation after 24h challenge by 10 µg/ml colistin. Overview images taken with a 40× objective. Scale bars represent 100 µm.

To investigate the effect of biofilm architecture on antibiotic susceptibility we established flow chamber biofilms with *E.coli* in presence and absence of the de-repressed IncF plasmids (wildtype, *traD*, *traQ* and *traX*). It has been previously shown that the *tra* mutations lead to formation of biofilms with strikingly different spatial distribution [6]. The different biofilms were treated with colistin and the distribution of living and dead cells monitored. Interestingly, strains with the de-repressed IncF WT plasmid and the *traD* mutation forming biofilms with elaborate, rounded tower structures rising from the confluent cell layer, displayed regions of tolerant cells in the center of the biofilm micro-colonies after 24h challenge with 10 µg/ml colistin. Moreover, this subpopulation formation seemed to be more extensive in the *traD* mutant biofilm compared to the wild type F^+^ (Fig. 4A). The strains lacking the IncF plasmid or carrying the *traQ* and *traX* mutant plasmids formed biofilms with a less structured organization, and without any recognizable mushroom shaped microcolony structures. In these biofilms no observable tolerant subpopulation was detected (Fig. 4A, data not shown). To evaluate the differences in bacterial survival in the biofilms quantitatively, we analyzed the CLSM data with the computer program COMSTAT [30]. The analysis showed that approximately 35% of the cells survived in the biofilm formed by SAR18 F^+^(*traD*), while survival was significantly lower (P<0.0030) for SAR18 F^+^ biofilms, in which around 15% of the cells were still green after 24h colistin treatment. In the biofilm formed by SAR18 F^+^(*traX*) less than 5% of the cells were viable after colistin challenge which is significantly less than SAR18F^+^ (P<0.0061). If the AF504 F^+^(*traD*) were challenged with colistin in the flow chamber system an extensive tolerant subpopulation was observed while when the AF504 F^+^(*traX*) strain no subpopulation could be detected (data not shown).

### Induction of colistin tolerance is *basS* dependent

The development of the tolerant subpopulation can be due to a phenotypic change of the bacteria in the center of the large micro colonies or to a generic difference in biofilm permeability. We established flow chamber biofilms of *E.coli* AF504 F^+^ with additional chromosomal mutations in the *basR/basS* two-component regulatory system. There was no measurable effect of the *basS* mutation on biofilm structure, surface coverage and biomass as determined by COMSTAT analysis (data not shown). No tolerant subpopulation could be detected in the AF504 *basS* F^+^ biofilms after 24 h colistin treatment (Fig. 4B). COMSTAT quantification of the acquired images clearly showed a significant difference in the ratio between PI stained cells and GFP expressing cells in the AF504 F^+^ and the AF504 *basS* F^+^ (data not shown) (P<0.0093). These results suggest that subpopulation formation is genetically regulated.

### The *yfbE* gene is induced within structured biofilms prior to colistin challenge

**Figure 5:**
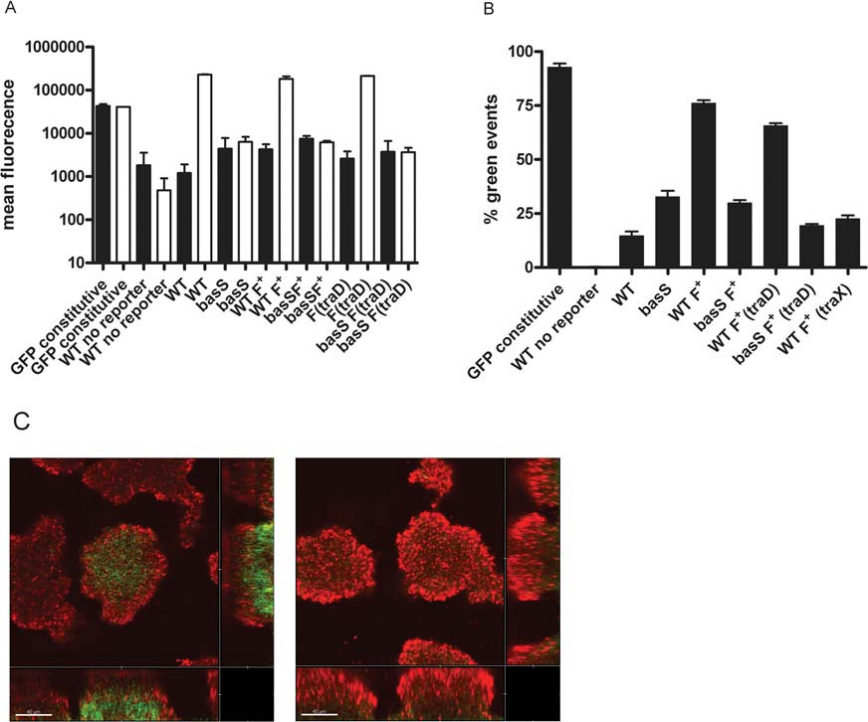
The *yfbE* gene is induced within structured biofilms prior to colistin challenge. A). Fluorescence of *yfbE*-*gfp* transcriptional fusion expressed in bacteria grown in N-minimal media, pH 7.7, containing 38 mM glycerol with either 10 mM Mg^2+^ (black bars), or 10 µM Mg^2+^ and 100 µM Fe^3+^ (white bars) Data shown are the average of three experiments. Mean fluorescence units were detected by using a Becton Dickingson FACS Vantage SE cell sorter. Strains harboring the *attB*::*bla-rrnB*P1::*gfp*mut3b or the pJBA113 vector alone demonstrated constitutive fluorescence and no fluorescence in all growth conditions, respectively. B). Fraction of fluorescent cells in the microtitre plate biofilm assay with the parental *E. coli* AF504 in the presence and absence of the de-repressed IncF plasmid and mutants in the IncF plasmid (*traD,*), and the reciprocal insertion deletions of the *basS* and *basR* genes, harboring the *yfbE*-*gfp* transcriptional fusion. The threshold for GFP fluorescence was cells with a value above 600 arbitrary units. 99% of the constitutively expressing cells were above this threshold while 96% of the negative control was below. C). CLSM micrographs showing the expression of the *yfbE*-*gfp* transcriptional fusion in flow chamber biofilms of AF504/F^+^ (left panel) and AF574 *basS/*F^+^ (right panel). Green indicates GFP expressing cells all other cells appear red from treatment with SYTO62.

The *ybf* operon consists of seven genes (*yfbE, F, G, H, arnT, yfbW* and *J*) that are implicated in the process of addition of the 4-amino-4-deoxy-L-arabinose (L-Ara4N) moiety to the phosphate groups of lipid A leading to increased polymyxin tolerance [32]. The induction of the *ybf* genes in vitro by low Mg^2+^ and high Fe^3+^ is under the control of the *basR/basS* two-component system [33], [34]. To determine if tolerant subpopulation formation is correlated with increased *yfbE* operon expression, we constructed a plasmid harbouring the AF504 *yfbE* promoter in front of a promoterless *gfp* gene and introduced it into our collection of AF504 strains with differential biofilm formation ability. Expression of the *yfbE::gfp* reporter was induced when the strains were grown in N-minimal media with 10 µΜ Mg^+2^ and 100 µM Fe^+3^ relative to the expression levels when the strains were grown in media with 10 mΜ Mg^+2^(Fig. 5A). No increase in fluorescence could be detected in strains, with a mutated *basS* gene, even when they were cultivated under inducing conditions (Fig. 5A), and no induction of the *yfbE* reporter could be detected by the addition of colistin to the N-minimal media under low or high Mg^2+^ conditions (data not shown). We then investigated GFP fluorescence of the different strains in microtiter biofilm assays. The bacterial population in the microtiter wells is highly heterogeneous and a direct measurement of the total fluorescence in the wells can be misleading. Instead, we determined the proportion of cells in the wells with elevated GFP fluorescence by using a strain with constitutive GFP expression to determine maximum range fluorescence and a strain with a promoterless vector control to determine minimum fluorescence. The proportion of induced fluorescent cells was significantly elevated in the biofilms formed by the AF504 F^+^ and AF504 F^+^(*traD*) strains compared to the biofilms formed by the wild type AF504 strain and the F^+^(*traX*) mutant (Fig. 5B). Interestingly, no increased fluorescence was detected in strains harboring a *basS* mutation indicating that observed increased fluorescence in the F^+^ and F^+^(*traD*) strains is due to elevated *yfbE* expression, and that the induction occurs prior to colistin challenge (Fig. 5B).

The *yfbE::gfp* reporter expression was also investigated in the hydrodynamic flow chamber system. AF504 F^+^ and AF504 *basS* F^+^ strains were inoculated in the system and GFP expression monitored after 24h our incubation without colistin treatment. Green fluorescence could be detected in the centre of large micro colonies in AF504 F^+^ biofilm while no fluorescence could be detected in *basS* mutant biofilm indictaing that there is a correlation of *yfbE* expression and sub population formation in flow chamber system (Fig. 5C).

## Discussion

The formation of bacterial biofilms is often associated with a reduced susceptibility to antimicrobial challenge [35], [36]. The mechanisms underlying the observed increased tolerance are poorly elucidated and a direct causal relationship between antibiotic tolerance development and biofilm formation is inadequately supported [37], [38]. Recently, Mah *et. al.* discovered that periplasmic glucans interact with the aminoglycoside tobramycin in *P. aeruginosa* biofilms and were proposed to delay by sequestration the effect of the antibiotic [39]. *P. aeruginosa* also has the ability to convert to an antibiotic-resistant phenotype after antibiotic exposure with enhanced biofilm forming ability [40]. A large proportion of the studies investigating biofilm tolerance are comparisons between cultures grown under shaking conditions (planktonic growth) and a particular biofilm model system [36], [41]–[49]. The result of this kind of comparisons can be misleading because there are many other factors affecting antibiotic susceptibility other than biofilm formation in these systems. Cell number, growth phase, and media flow are factors that are varying in the different growth systems and can potentially affect antibiotic tolerance. There is a risk that results obtained under these conditions more reflect differences between the particular growth model systems rather than actually describing particular aspects of biofilm physiology compared to planktonically grown cells. In studies where tolerance has been investigated under physiologically similar conditions the differences in susceptibility has been less dramatic [43], [50]. To better understand the influence of biofilm formation on the development of antibiotic tolerance, we exploited the recent discovery of Reisner *et al.* [6] that biofilm structure and organization can be manipulated by varying the IncF transfer pilus configuration (Fig. 1 A). Using this set of isogenic *E. coli* strains enable the investigation of antibiotic tolerance and resistance under nearly identical physiological conditions and the direct study of the influence of biofilm formation on antibiotic tolerance rather than comparing different growth model systems. We demonstrate that biofilm formation can fundamentally influence bacterial survival upon antibiotic challenge, but the increased survival did not correlate directly with biofilm forming ability as such, but rather depended on the organization of the biofilm and on the antibiotic used (Fig. 1 and 2). When the biofilm forming strains were challenged with the surface-active peptide antibiotic colistin, there was a dramatic difference in survival between strains harboring the de-repressed incF transfer pilus and the plasmid free strains (Figs. 1 and 2). In contrast, when the biofilms were challenged with the fluoroquinolone ciprofloxacin, an antibiotic with an intracellular target, no significant differences in survival were observed between the investigated strains. Interestingly, resistance to ciprofloxacin is only known to occur through chromosomal mutations in *E. coli,* while antimicrobial peptide resistance and tolerance can arise through both physiological and mutational mechanisms [20], [21], [34], [51]. In fact, our data suggest that physiological tolerance mechanism may be essential for the increased colistin tolerance of bacteria in biofilms.

When flow chamber biofilms with complex spatial organization were challenged with colistin, subpopulations of tolerant cells were observed, while no such populations were detected in biofilms with flat, undifferentiated structure (Figs. 3 and 4). The localization of the subpopulation in the center of the micro-colonies indicates that the local environment within the biofilm induces subpopulation formation in our experimental system. Tolerance development is thus structurally dependent, influenced by the conditions created within the biofilm. However, we do not believe our data directly support a correlation of the difference in colistin tolerance to a distinct spatial organization and it is important to make distinction between a phenotype being structurally dependent and being correlated with a specific structure. When mutations in *basS* were introduced into the strains with wildtype F as well as the *traD* mutant, survival was comparable to the strain without F (Fig. 2A). Since *basS* is regulating the LPS modifications leading to augmented antimicrobial peptide tolerance, these data show that the increased survival is directly coupled to a genetically regulated event. We show that the expression of the *basR/S* regulated *yfbE* operon is up-regulated in the strains with increased survival and subpopulation formation, when the strains are grown as biofilms (Fig. 5B). Because the enzymes encoded by the operon are involved in the process of LPS modification, it is likely that it is through this process that the tolerant subpopulation is formed [32]. The increased expression of the operon occurs prior to colistin addition, indicating that the subpopulation formation process is not directly coupled to the addition of colistin or an adaptation to the presence of antimicrobial peptide (Fig. 5B). The main rational behind including the *basRS* mutant was to be able to differentiate between the development of a generic biofilm induced tolerance and specific genetic regulation to explain the observed increased survival in the F^+^ and F(*traD*) biofilms. Our data cannot rule out that there are other *basRS* dependent resistance mechanisms than LPS modification involved in the observed phenomena. This possibility does not change the fact that colistin tolerance is dependent on a specific genetic mechanisms rather than a generic biofilm property.

When *P. aeruginosa* was challenged with colistin in the flow chamber system, a tolerant subpopulation was also found [52]. In contrast to *E. coli*, the surviving population was not observed in the center of large micro colonies but rather on the surface of the biofilm, and it was associated with a migrating cell population that eventually colonized the top of the microcolonies, resulting in the development of mushroom-shaped multicellular structures characterizing *P*. *aeruginosa* flow chamber biofilms [52]. The *P. aeruginosa pmr* system is known to be activated by the presence of cationic antimicrobial peptides [53], [54]. The *yfbE* homolog, *pmrH*, was indeed induced in the migrating tolerant subpopulation when the peptide was added to the system while cells in the stalks of the mushroom-shaped micro colonies seemed unable to induce or sense the presence of the peptide and were subsequently killed [52]. It is also interesting to note that even though the cells in the deeper layers of the flow chamber biofilm were coated by a thick layer of tolerant cells, this was not able to protect these cells from the action of the antibiotic [52]. No colistin-mediated induction has been shown for *E.coli,* indicating a dissimilar regulation of homologous genes responding to disparate environmental stimuli since a migrating population is not present in the IncF mediated biofilm. Even though the tolerant subpopulations form at different locations within the *E.coli* and *P.aeruginosa* biofilms, there are notable common features. Both populations require the presence of genetically encoded tolerance mechanisms and seem to be influenced by specific environmental cues that are independent of general biofilm properties (Figs. 3 and 4) [52].

Physicochemical gradients have previously been demonstrated in microbial biofilms [9], [10]. However, little is known about how these gradients affect the behavior of the cells integrated in the biofilm structure. The specific signals regulating the formation of the colistin tolerant subpopulation in *E.coli* are currently unknown, but since the development is under control of the *basR/basS* two-component system, it is probable that local environmental stimuli affect the cells in the biofilms. It is known that media with low Mg2+ in combination with high Fe^3^+ ion concentrations activate this regulatory network *in vitro* (Fig. 5B) [34]. We find it unlikely that such conditions prevail inside the large micro-colonies under the conditions tested. Luria broth is a medium with relatively low Mg2+ levels, but no extra iron is added in the experiments making it difficult to imagine a local accumulation of this ion in the micro colonies in sufficient amounts to induce *basS* activation. The magnesium ion concentration in the minimal medium used in flow chamber experiments is sufficiently high to prevent *basS* dependent induction, even in high iron conditions in a non biofilm setting and it is therefore unlikely that differential magnesium iron concentration is responsible for the observed induction [33]. The close proximity of the cells in the larger micro colonies in biofilm communities may create conditions mimicking the *in vitro* effect of high iron levels. The bacteria may have to undergo complex physiological adaptations in response to the consequences of the high cell density within the micro-colonies. It is interesting to note, that the homologous operon to the *yfbE*, the *pmrH* operon in *Salmonella typhimurium* is induced during swarm-cell differentiation, and an increased tolerance of swarmer cells to peptide antibiotics have been found to be due to an altered phenotypic state of the cells, in the absence of genetic changes [55], [56]. It is possible that the conditions within the organized microcolonies mimic those that induce swarm-cell development. In addition, it cannot be ruled out that the *basR/basS* are components of a general stress response to toxic compounds produced by the metabolism of the cells in the micro-colonies.

The data presented here suggest that biofilm mediated antibiotic tolerance may often be a matter of subpopulation differentiation within the biofilm. The basis for tolerance development is not the biofilm formation *per se*, but that the spatial and environmental heterogeneity provided by structurally organized biofilms have the capacity to promote tolerant subpopulation formation. In our experimental system, the increased antibiotic tolerance exhibited by biofilm formation is antibiotic specific and conditional, dependent on the actual biofilm structure and on the presence of a specific genetically encoded tolerance mechanism. Our data even suggest that specific modes of biofilm organization may induce antibiotic tolerance, and that the factors determining the properties of heterogeneous aggregated microbial communities may act on the individual cells within the structures rather than on the community. No heritable genetic changes are involved in the phenomenon; we suggest that many other cases of biofilm-induced tolerance to antibiotics could be the result of similar mechanisms. The biofilm mode of growth does not directly predict antibiotic resistance and we suggest that a complete elucidation of bacterial biofilm antibiotic tolerance phenomena merits investigations that are not limited by preconceived assumptions of the consequence of biofilm formation.

## Materials and Methods

### Bacterial strains, plasmids and growth conditions

The bacterial strains relevant to this study are listed in Table 1. Plasmids and PCR primers used in the study are listed in Table 2. Except where indicated, the strains used are derivatives of *E. coli* K-12 strain MG1655 or CSH26. Luria–Bertani (LB) broth or agar [54] was used for standard cultivation supplemented with 0.2% glucose. Appropriate antibiotics were added at the following final concentrations 100 µg ml^−1^ ampicillin (Amp), 10 µg ml^−1^ chloramphenicol (Cm), 100 µg ml^−1^ nalidixic acid (Nal) and 50 µg ml^−1^ kanamycin (Km) when required.

**Table 2:** Plasmids and primers used in this study.

Plasmids	Description	Reference
pAR108	pOX38Km *aph*::cat-PA1/04/03-cfp*	[6]
pOX38-*traD411*	pOX38 *traD:: kan*	[64]
pOX38-*traQ238*	pOX38 *traQ::kan*	[65]
pOX38-*traX482*	pOX38 *traX::kan*	[24]
pSK410	F *traD* in pMS470Δ8	[66]
pSM1690	pLOW2 NotI-*rrnB*P1::*gfp*mut3b*	[31]
pKD3	Chloramphenicol insertion template.	[29]
pKD4	Kanamycin insertion template.	[29]
pKD46	Red recombinase encoding plasmid	[29]
pLDR8	*int* gene expression helper vector	[59]
pLDR11	Cloning vector for *attB* integration	[59]
pJBA113	Apr,pUC18Notl-PA1/04/03-RBSII-*gfp*(ASV)-T0-T1*a*	[67]
pYFBe32	Derivative of pJBA113 carrying *PyfbE* PCR fragment	This work
pBASrs2	Derivative of pJBA113 carrying *basRS* fullength PCR fragment	This work

Primers	Description	Reference
basSmutf	TCCCCGCATTTACTCTCATCAATACCTGGTCCTTCAGTGTAGGCTGGAGCTGCTTC	This work
basSmutr	TCTGCGCCGACCAATATCGCTGCGCCAACGGCTGATATGGGAATTAGCCATGGTCC	This work
ecoBasR1	ATATAAGAATTCCTGTGGAATGACAACGATGG	This work
xbaBasS1	AATTAATCTAGAAATATTTCGGTGCTGGTTGC	This work
yfbEeco	AATTAAGAATTCGAGCGGCAAAAAGCTAACAC	This work
yfbExba	ATATAATCTAGAGGGAAGGCGTAATCACTTCA	This work

### Genetic techniques and mutant construction

Unless otherwise stated all genetic methods followed standard procedures as described by Ausubel, F.M., and others [53]. *E. coli* phage P1 transductions were performed by the method of Miller [57]. To enable conjugative plasmid transfer a spontaneous nalidixic acid resistant mutant of MG1655 was isolated on LB agar plates containing 10 µg ml^−1^ nalidixic acid at 37°C and designated AF504. Conjugations of pAR108 pOX38-*traD411* and pOX38-*traX482* into AF504 from were performed by plate mating [58] with SAR18/pAR108, SAR18/pOX38-*traD411* pSK410 and SAR18/pOX38-*traX482* as respective donors. The defined insertion mutations in *basS* and *basR* were produced by using one-step allelic replacement utilizing the λ Red recombinase system [29]. In short, transformants of AF504 carrying the Red recombinase plasmid pKD46 were grown in 5 ml SOB cultures with ampicillin and L-arabinose at 30°C. pKD46 shows temperature-sensitive replication and the Red recombinase genes are under the control of a arabinose sensitive promoter. The culture was grown to an OD600 of approximately 0.6 and then made electro-competent by washing four times in ice-cold 15% glycerol. PCR products were polymerase chain reaction (PCR)-amplified via the Expand High Fidelity System (Roche) on a Biometra T3 thermocycler using the plasmids pKD4 and pKD3 as templates for kanamycin resistant and chloramphenicol resistant insertions respectively. The amplified products were gel-purified, digested with *DpnI*, repurified and suspended in water. Electroporation was performed by using a Gene pulser (Bio-Rad) as recommended by the manufacturer using 50 µl bacterial cells and 10–100 ng PCR product. The cells were then added to 1 ml of SOC medium [53] and incubated for 1 h at 37°C and then one-half was spread onto selective agar plates. After primary selection, putative mutants were re-streaked on selective media at 37°C and then tested for ampicillin sensitivity. Putative insertion mutations were transduced with phage P1 into fresh AF504 background for further phenotypic characterization. The primers basSmutf and basSmutr were used for allelic replacement. Insertions were verified by PCR. The cloning of the full-length *basRS* genes and *yfbE* promoter region, the primers ecoBasR1, xbaBasS1 and yfbEco, yfbExba were used, respectively. The primers contain an EcoRI restriction site or an XbaI for site to facilitate insertion in the corresponding restriction sites on the *gfp* expression vector pJBA113. The amplification was performed as described above for the mutagenesis PCR. The PCR fragment was inserted into the corresponding sites in the multiple cloning site of plasmid pJBA113 and electroporated into *E. coli* XL10 (Stratagene), resulting in the transcriptional GFP reporter plasmids pBASrs and pYFBE32. DNA extraction, treatment with modifying enzymes and restriction endonucleases, ligation of DNA fragments with T4 ligase and transformation of *E. coli* was performed using standard methods [53]. Plasmid DNA was isolated with the Spin Miniprep Kit (Qiagen). DNA fragments agarose gel purified using GFX DNA Purification system (Amersham Pharmacia Biothech). Insertion of gfpmut3* under the *rrnB*P1 promoter in the *attB* site *E.coli* AF504 was performed using a vector system previously described [59]. The pLDR11 vector was digested with NotI endonuclease, dephosphorylated and the 1.8 kb fragment containing the *bla* gene and the lambda *attP* attachment site was isolated. This fragment was ligated with a NotI fragment of pSM1690. Competent *E. coli* cells carrying transformants were selected at 42°C.

### Microtitre plate biofilm and antibiotic tolerance assays

Biofilm formation and antibiotic tolerance was quantified using the static microtiter plate biofilm assay. Bacterial cultures of the investigated strains were grown in 5ml LB in 37°C to turbidity. The cultures were diluted 1:300 and inoculated in triplicate in a 96-well microtiter plate (V-bottom; BioSterilin). Test plates were transferred to plastic bags to avoid evaporation and statically incubated for 24 h at 37°C. Biofilm formation was assayed by staining of attached cells with crystal violet as described [60]. In short, the growth medium was carefully removed from the biofilm microtiter wells, washed twice with 0.9% NaCl solution, and covered with 0.1% CV for 15 min. The wells were again washed twice with 0.9% NaCl where after surface bound CV was extracted by addition of 96% ethanol. Absorbance of stained wells was determined at 590 nm with a VICTOR^2^ multilabel counter (Perkin Elmer, Inc.). For the antibiotic tolerance microtiter assay, biofilms were grown in microtitre plates without antibiotics as for biofilm quantification. After the 24 h incubation, colistin (H. Lundbeck, Copenhagen) or ciprofloxacin (Bayer) was carefully added to the wells at the indicated concentrations. After 24 h static additional incubation cells were vigorously re-suspended and the total CFUs were determined by serial dilution and plating on LB plates without prior washing. Consistent removal of the bacterial cells was confirmed by crystal-violet staining of the wells. For microtitre biofilm assays and tolerance assay the data is presented as mean±S.E.M. of 3 experiments performed in triplicate. In parallel with each biofilm experiment, a microtiter plate MIC was determined for the planktonic cells in the culture used as the inoculum. That the biofilm-associated cells truly were dispersed to single cells by vigorous aspiration was ascertained by observing the dislodged biofilm under a microscope prior to plating.

### GFP Expression Assay and *yfbE* expression visualization

Strains harboring the plasmids pYFBe32 were grown in N-minimal media, pH 7.7, containing 38 mM glycerol with either 10 µM MgCl_2_, or 10 µM MgCl_2_ and 100 µM FeSO_4_ and supplemented with 50 µg/ml ampicillin. GFP expression was analyzed after 6h of growth at 37°C by using a fluorescence-activated cell sorter (Becton Dickinson FACS Vantage SE). Assays were performed in triplicate. For *yfbE* expression in biofilms, strains were inoculated in 96 well microtiter plates as described for the antibiotic tolerance assay. After incubation for 24 h at 37°C cells were vigorously re-suspended washed twice in 0.9% NaCl_2_ and GFP fluorescent monitored in the FACS. Strains harboring the *attB*::*bla-rrnB*P1::*gfp*mut3b (GFP constitutive) and the pJBA113 vector alone (WT no reporter) demonstrated constitutive fluorescence and no fluorescence in all growth conditions, respectively. The threshold for GFP fluorescence was set as cells with a value above 600 arbitrary units. 99% of the constitutively expressing cells were above this threshold while 96% of the negative control was below. Experiments were done in triplicate. For visualization of *yfbE* expression in the hydrodynamic flow chamber systems the non-GFP fluorescent cells were counterstained with SYTO62 (Invitrogen) according to the manufacturers instructions.

### Bacterial viability assay and fluorescence-activated cell sorting

The LIVE/DEAD *Bac*light bacterial viability kit (Invitrogen) was used to for distinguishing live and dead bacteria after antibiotic treatment according to the manufacturers instructions. For the biofilm experiments fluorescence-tagged bacteria (GFP) and red-fluorescent propidium iodide (PI) staining were used. Red fluorescing and green fluorescing cells from a colistin-treated biofilm were sorted by fluorescence-activated cell sorting (Becton Dickinson FACS Vantage SE) and plated on Luria Bertani plates to verify that red fluorescing cells were not viable and green fluorescing cells were viable.

### Flow chamber biofilms

Cultivation of biofilms in laminar flow was done at 30°C as previously described [6]. Flow channels irrigated with AB minimal medium [61] supplemented with 0.01 mM Fe-EDTA, 1.0 mg/ml thiamine,or 1.0 mg/ml proline and uracil, where appropriate. The channels were inoculated with 250 µl of normalized dilutions (optical density at 600 nm of 0.05) of saturated bacterial cultures. After 24h incubation, biofilms were challenge with 50 µg/ml, 25 µg/ml or 10 µg/ml colistin for an additional 24h where after the bacterial red fluorescent viability stain propidium iodide was injected into the flow channels and incubated for 15 min.

### Images acquisition and analysis

All microscopic observations were performed by a Zeiss LSM510 Confocal Laser Scanning Microscope, CLSM (Carl Zeiss, Jena, Germany) equipped with an argon/krypton laser and detectors and filter sets for simultaneous monitoring of GFP (excitation 488 nm, emission 517 nm) and red-fluorescents emitted from the PI (excitation 543nm, emission 565 nm). For the quantification of biofilms and analysis of living and dead cells in treated biofilms the software COMSTAT was used [62]. Images were obtained using a 40×/1.3 Plan-Neofluar oil objective. Multichannel simulated fluorescence projection (SFP, a shadow projection) images and vertical cross sections through the biofilm were generated by using the IMARIS software package (Bitplane AG, Zürich, Switzerland) running on a PC. Images were further processed for display by using Photoshop software (Adobe, Mountain View, Calif.). Time series experiments were performed on the Zeiss LSM510 microscope and movie sequences were produced using the Jasc software, Animation Shop 3. For the quantification of *E. coli* F+ wild type and the different mutants three independent biofilm experiments were performed acquiring at least 9 image stacks for each strain and situation/time point randomly down through the flow channel using a 40x/1.3 Plan-Neofluar oil objective. For the quantification of living and dead cells, each strain was grown in three channels in three independent experiments, and from each experiment six image stacks were acquired randomly in the channel. Images were further treated using COMSTAT [62].

### Statistical analysis

Statistical significance was determined using unpaired Student's *t*-test, values of *P*<0.05 were considered significant.

## Supporting Information

Movie S1 *E.coli* flow-chamber biofilms are sensitive to the cationic antimicrobial peptide colistin. Time series showing killing dynamic of a mature AF504 *gfp*/F^+^ biofilm. Staining of the biofilm with PI (red) before/during image acquisition for monitoring of dead cells. Images were acquired every 15 min for 42 h.
(7.65 MB MPG)
